# Optimizing Ultrasensitive RT‐QuiC assay on AD tau seed‐monomer reactions

**DOI:** 10.1002/alz.088219

**Published:** 2025-01-03

**Authors:** Junle Chen, Virginia M.‐Y. Lee, Hong Xu

**Affiliations:** ^1^ University of Pennsylvania, PHILADELPHIA, PA USA; ^2^ Center for Neurodegenerative Disease Research, PHILADELPHIA, PA USA; ^3^ Institute on Aging, University of Pennsylvania, Perelman School of Medicine, Philadelphia, PA USA; ^4^ Center for Neurodegenerative Disease Research, Department of Pathology and Laboratory Medicine, University of Pennsylvania, Perelman School of Medicine, Philadelphia, PA USA; ^5^ University of Pennsylvania, Philadelphia, PA USA

## Abstract

**Background:**

Alzheimer’s disease (AD) is pathologically defined by the presence of extracellular Aβ plaque and intracellular tau inclusions. Emerging evidence shows that tau aggregates contain pathogenic bioactivities of templating monomeric tau into filamentous fibrils and propagating through cells. Based on these findings, assays have been developed to detect minute amounts of pathogenic tau in human samples. With great potential, it remains unclear about the sensitivity and fidelity of tau fibrillization due to the presence of pathogenic tau species.

**Methods:**

To that end, our study focuses on optimizing the Real‐Time Quaking‐Induced Conversion (RT‐QuiC) assay for its potential of detecting and amplifying insoluble tau seeds derived from AD patient. To this end, we utilized recombinant tau monomers t306 and k12, 2R and 3R tau peptide respectively known for their rapid fibrillization and high amplification signals in previous studies. More specifically, we optimized the RT‐QuiC assay using both crude and purified brain lysates from AD and control cases.

**Results:**

The specificity of the RT‐QuiC assay were successfully reproduced by multiple cases of AD and control brain lysates. Moreover, we tested the RT‐QuiC assay with purified AD tau seeds and assessed the sensitivity of the assays based on the amount of insoluble tau. To further interrogate the system, we also evaluated the impact of reaction buffer on the ThT‐based measurement of tau fibrillization. Our results show that the original RT‐QuiC assay can sensitively detect insoluble AD tau seeds at the nM level in our hands. The optimized RT‐QuiC assay could differentiate better between AD‐seeded reactions and controls with an improved sensitivity up to fM level, with a better separation between AD and control and faster reaction rate. The assay showed a clear dose‐dependent response till pM range.

**Conclusion:**

Upon optimization, the RT‐QuiC assay is both ultrasensitive and specific to AD tau fibrillization. Our findings offer insights into the fibrillization mechanism of tau aggregates and expand our understanding of pathogenic tau seeds in AD. By optimizing the assay to more accessible patient samples in the future, namely CSF and plasma, the RT‐QuiC can serve as a potent diagnostic tool for AD tau progressions.

